# Structural and functional evolution of IL-1 targeting: From systemic neutralization to bioengineered nanotherapies

**DOI:** 10.1016/j.mtbio.2026.103057

**Published:** 2026-03-25

**Authors:** Lan Yang, Guodong Chen, Zhengbing Lyu

**Affiliations:** a College of Life Sciences and Medicine, Zhejiang Provincial Key Laboratory of Silkworm Bioreactor and Biomedicine, Zhejiang Sci-Tech University, Hangzhou, 310018, China; b Center for Medical Genetics, School of Life Sciences, Central South University, Changsha, 410013, China; c Zhejiang Sci-Tech University Shaoxing Academy of Biomedicine Co.,Ltd, Shaoxing, 312369, China; d Furong Laboratory, Xiangya School of Medicine, Central South University, Changsha, 410013, China

**Keywords:** Interleukin-1, Antibody, Variable new antigen receptor, Structure-function coevolution, Targeted therapy

## Abstract

The Interleukin-1 (IL-1) superfamily mediates critical inflammatory responses. While monoclonal antibodies targeting IL-1α and IL-1β have transformed the management of autoinflammatory, rheumatologic, and cardiovascular disease, their efficacy is often limited by poor tissue penetration, compensatory cytokine networks, and systemic immunosuppression risks. This Review traces the structural and functional evolution of IL-1 therapeutics from early polyclonal detection to modern high-affinity systemic neutralization. We examine the biophysical barriers restricting conventional immunoglobulins within necrotic and fibrotic microenvironments and discuss the emerging paradigm of dual-blockade bispecifics and combinatorial immunotherapies. Finally, we highlight the transition toward a "Bioengineering Era." By leveraging the superior diffusion kinetics of single-domain antibodies—such as camelid nanobodies and shark-derived VNARs—and integrating them with stimuli-responsive hydrogels and theranostic nanocarriers, we outline a paradigm shift from broad systemic blockade to precision, tissue-specific immunomodulation.

## Highlights

The functional dichotomy between IL-1α (a local alarmin) and IL-1β (a systemic pyrogen) necessitates context-specific therapeutic strategies.
While conventional monoclonal antibodies transformed treatment, their efficacy in fibrotic microenvironments is restricted by poor diffusion and systemic immunosuppression risks.
Single-domain antibodies, including camelid nanobodies and shark VNARs, overcome these biophysical barriers by providing superior tissue penetration and access to cryptic epitopes.
To counter compensatory cytokine networks, dual-blockade bispecifics and combinatorial immunotherapies are emerging as robust therapeutic alternatives.
Integrating nanobodies into ROS-responsive hydrogels and theranostic nanocarriers shifts the paradigm from broad systemic neutralization toward on-demand, localized precision immunomodulation.

## Graphical abstract

**Figure:**
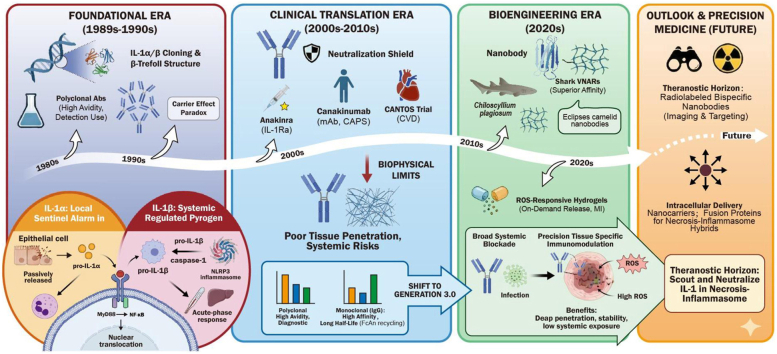

## 1 Introduction

The vertebrate innate immune system relies on rapid, non-specific responses to danger signals, a process centrally regulated by the Interleukin-1 (IL-1) superfamily. Since the initial molecular cloning of IL-1α and IL-1β in the 1980s, these cytokines have been recognized as the central regulators of inflammation. Encoded by distinct genes clustered on human chromosome 2q14, they share a low sequence homology (approximately 27% amino acid identity) yet possess a remarkable structural convergence, both folding into a β-trefoil architecture composed of 12 anti-parallel β-strands [1]. This structural conservation allows them to bind the same receptor complex, initiating a signaling cascade that is fundamental to survival yet highly pathogenic when dysregulated.

The clinical significance of modulating this pathway is highlighted by the wide spectrum of IL-1-driven pathologies. Unlike adaptive immune responses, which are antigen-specific, IL-1 signaling is a universal amplifier. It drives the articular destruction in rheumatoid arthritis (RA), the severe acute flares of gouty arthritis, the systemic autoinflammation of Cryopyrin-Associated Periodic Syndromes (CAPS), and the subclinical, chronic inflammation underlying atherosclerosis and type 2 diabetes [2]. Moreover, recent evidence implicates IL-1α in the remodeling of the tumor microenvironment (TME), promoting angiogenesis and metastasis in colorectal and lung cancers [3].

Therapeutic strategies have evolved in parallel with our understanding of cytokine biology. The early "Foundational Era" focused on characterizing natural antagonists (IL-1Ra) and developing polyclonal antibodies (pAbs) for detection. This laid the groundwork for the "Clinical Translation Era," where recombinant proteins (anakinra) and monoclonal antibodies (mAbs) validated the therapeutic hypothesis in humans [4]. Currently, the field is transitioning into the "Bioengineering Era." As identified in recent literature, the field is shifting towards single-domain antibodies (sdAbs) and smart biomaterials designed to overcome the pharmacokinetic limitations of conventional IgGs [5,6].

**Fig. 1:**
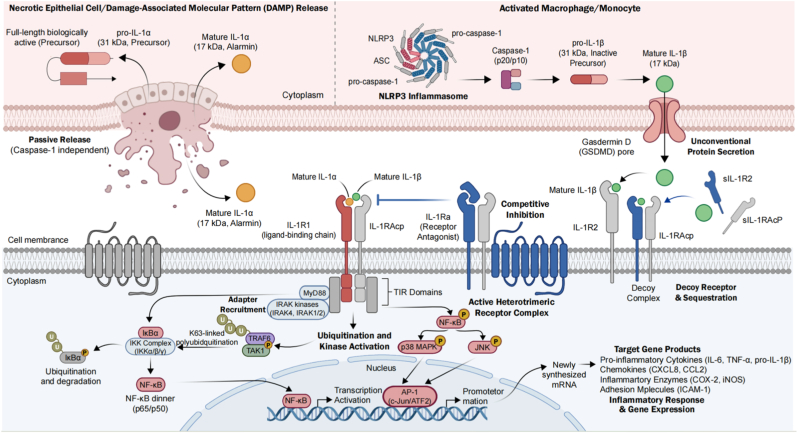
**Molecular mechanisms of IL-1α and IL-1β signaling, regulation, and downstream effector pathways.**IL-1 isoforms utilize distinct release mechanisms but convergent signaling. Left: Bioactive precursor IL-1α is passively released from damaged barrier cells as an alarmin. Right: IL-1β requires priming and caspase-1-dependent cleavage by the NLRP3 inflammasome for secretion from immune cells. Both bind the common IL-1R1/IL-1RAcP complex. Signaling is inhibited by IL-1Ra and IL-1R2. Receptor activation recruits MyD88, triggering downstream cascades that result in NF-κB and AP-1 nuclear translocation and proinflammatory gene expression.

**Fig. 2:**
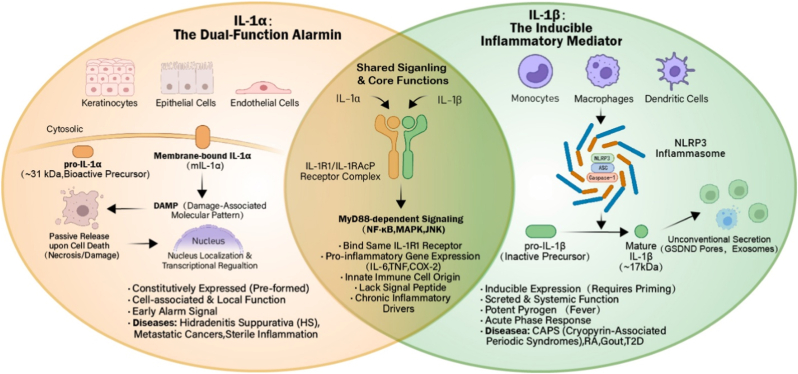
**Comparative analysis of distinct and conserved features of IL-1α and IL-1β.**IL-1α (left) is primarily derived from epithelial and endothelial sources, acting locally as a cell-associated or passively released alarmin in sterile inflammation. IL-1β (right) is produced by myeloid cells and requires regulated inflammasome-mediated processing for active secretion to function as a systemic pyrogen. Overlap (center): Both isoforms lack a canonical signal peptide, utilize the shared IL-1R1 signaling complex, and drive chronic inflammatory pathologies.

**Fig. 3:**
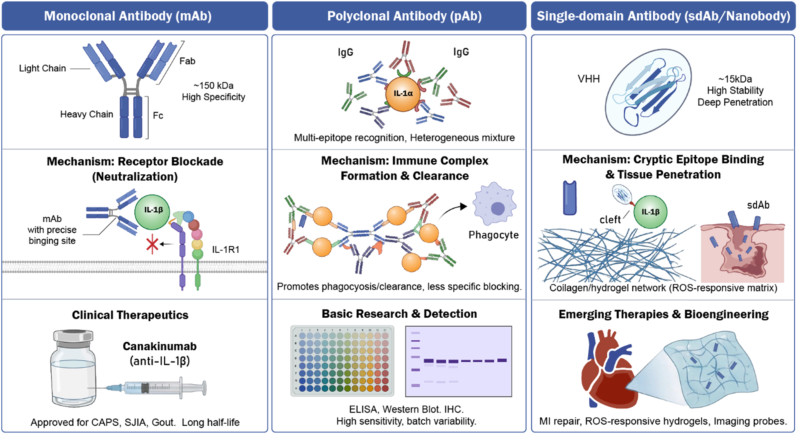
**Structural characteristics, mechanisms, and applications of IL-1 antibody modalities.**Left (mAb): Full-length IgGs (∼150 kDa) provide high specificity and long serum half-lives. Mechanism involves steric blockade (neutralization) of cytokine-receptor binding, forming the basis for clinical therapeutics (e.g., canakinumab). Middle (pAb): Heterogeneous mixtures recognizing multiple epitopes. They form large immune complexes to promote clearance, utilized primarily for sensitive laboratory detection techniques (ELISA, Western Blotting). Right (sdAb/Nanobody): Single stable VHH domains (∼15 kDa). Their small size enables deep tissue penetration and access to cryptic epitopes, suited for emerging bioengineering applications like targeted delivery for tissue repair.

**Fig. 4:**
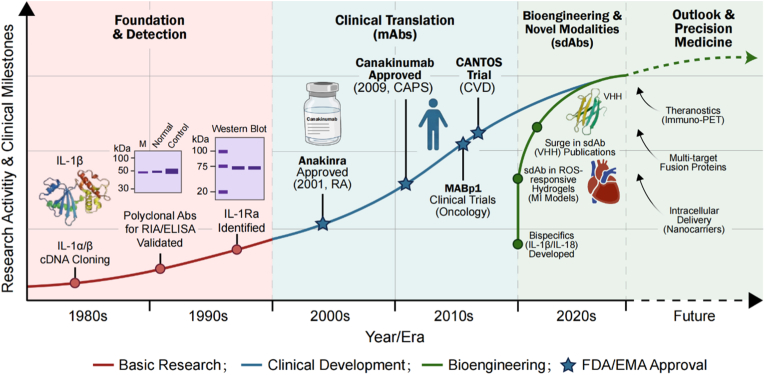
**Milestones in IL-1 research and therapeutic development across technological eras.**Foundational Era (1980s-1990s, red): Focused on molecular cloning of IL-1α/β, establishing pAb-based detection methods, and discovering the natural antagonist IL-1Ra. Clinical Translation Era (2000s-2010s, blue): Marked by regulatory approval of therapeutics including recombinant IL-1Ra (anakinra) and mAbs (e.g., canakinumab), validating IL-1 blockade in inflammatory diseases. Bioengineering Era (2020s-present, green): Emphasizes novel modalities (sdAbs, bispecifics) for applications in tissue engineering and regenerative medicine. Future outlook projects advancements in precision medicine via theranostics and intelligent delivery systems. (For interpretation of the references to color in this figure legend, the reader is referred to the Web version of this article.)

This review provides a comprehensive analysis of this evolution. We begin by dissecting the divergent release mechanisms of IL-1α and IL-1β (Fig. 1, Fig. 2), establishing the biological rationale for distinct therapeutic targets. We then contrast the structural modalities of available antibodies—pAbs, mAbs, and sdAbs (Fig. 3)—analyzing how their biophysical properties dictate their clinical utility. To address the limitations of conventional monotherapies, we further evaluate the recent paradigm shift toward dual-blockade bispecific antibodies and synergistic combinatorial strategies. Finally, tracking historical and future milestones (Fig. 4), we explore the cutting-edge integration of nanobodies into reactive oxygen species (ROS)-responsive hydrogels and theranostic nanosystems, highlighting a transition from broad systemic neutralization toward precision, localized immunomodulation.

## 2 Spatiotemporal regulation defining the alarmin and pyrogen dichotomy

To engineer effective therapeutics, one must first appreciate that while IL-1α and IL-1β converge on the same receptor, their upstream regulation is fundamentally distinct. This dichotomy represents the first layer of specificity in therapeutic design.

### 2.1 Distinct cellular origins and divergent release mechanisms

As illustrated in Fig. 1, Fig. 2, the two isoforms exhibit distinct spatiotemporal profiles during inflammation, dictated by their cellular sources and mechanisms of release.

#### 2.1.1 IL-1α: The sentinel alarmin

IL-1α is constitutively expressed in a wide variety of non-hematopoietic barrier cells, including keratinocytes, epithelial cells of the gastrointestinal and respiratory tracts, and endothelial cells [7,8]. Unique among interleukins, the precursor form of IL-1α (pro-IL-1α, ∼31 kDa) is fully bioactive [9]. It contains a nuclear localization signal (NLS) within its N-terminal pro-domain, allowing it to shuttle between the cytosol and the nucleus, where it acts as a transcription factor regulating cell growth and differentiation [10].

In the context of pathology, IL-1α functions primarily as an "alarmin" or Danger-Associated Molecular Pattern (DAMP) [[11], [12], [13]]. As depicted in Fig. 1 (Left) and Fig. 2 (Left), release of IL-1α is often a passive event triggered by loss of membrane integrity [[14], [15], [16]]. During ischemic necrosis—such as that seen in myocardial infarction, stroke, or the necrotic core of solid tumors—pro-IL-1α is passively released into the extracellular milieu [17,18]. Because it requires no enzymatic processing to bind its receptor, it initiates an immediate inflammatory response, recruiting neutrophils to the site of sterile injury within minutes [19]. This binary activation mechanism is dependent solely on cell death, making IL-1α a critical marker of tissue damage rather than infection. Furthermore, a membrane-bound form of IL-1α on activated monocytes allows for juxtacrine signaling, enabling immune cells to stimulate adjacent fibroblasts or endothelial cells through direct contact.

#### 2.1.2 IL-1β: The regulated pyrogen

By contrast, IL-1β is not constitutively expressed in health. It is the product of hematopoietic cells—monocytes, macrophages, and dendritic cells [7,8]—and its production is governed by a rigorous "two-signal" checkpoint system designed to prevent accidental activation (Fig. 1, Right; Fig. 2, Right).

• **Signal 1 (Priming):** The sensing of Pathogen-Associated Molecular Patterns (PAMPs) via Toll-like Receptors (TLRs) leads to the activation of NF-κB, inducing the transcription and translation of the inactive precursor, pro-IL-1β [20]. Unlike pro-IL-1α, pro-IL-1β is biologically inert and unable to bind the IL-1 receptor [21,22].
• **Signal 2 (Activation):** A second stimulus—such as ATP, pore-forming toxins, or crystalline particulates like monosodium urate (MSU)—triggers the assembly of the NLRP3 inflammasome [[23], [24], [25]].

The inflammasome, a cytosolic oligomer, recruits the adaptor protein ASC and pro-caspase-1 [[26], [27], [28]]. The subsequent auto-proteolysis of caspase-1 generates the active enzyme, which cleaves pro-IL-1β into its mature, 17 kDa bioactive form [29]. Once secreted, it acts systemically, circulating to the hypothalamus to induce fever and to the liver to trigger the acute-phase response [30,31](e.g., CRP production). This stringent regulation ensures that the potent systemic effects of IL-1β are restricted to bona fide pathogenic stimuli.

### 2.2 Signaling pathways and regulatory checkpoints

Despite their divergent origins, both cytokines utilize the same signaling apparatus (Fig. 1, Center). Upon binding to the primary receptor, IL-1R1, the complex undergoes a conformational change that recruits the IL-1 Receptor Accessory Protein(IL-1RAcP) [32,33]. This trimerization brings the intracellular Toll/IL-1 Receptor (TIR) domains into proximity, serving as a scaffold for the recruitment of the adaptor molecule MyD88 [[34], [35], [36]].

The assembly of the "Myddosome" triggers a serine/threonine kinase cascade involving IRAK4, IRAK1/2, and TRAF6 [37]. This pathway bifurcates to activate the Mitogen-Activated Protein Kinases (MAPK), including p38 and JNK, and the IκB kinase (IKK) complex [38]. The latter phosphorylates the inhibitor IκBα, marking it for proteasomal degradation and releasing the transcription factor NF-κB (p65/p50) to translocate to the nucleus [39]. The result is the robust expression of pro-inflammatory genes, including *IL6*, *TNF*, *COX2*, and adhesion molecules like *ICAM1*.

This signaling cascade is endogenously regulated via natural antagonists: the IL-1 receptor antagonist (IL-1Ra), which competes for IL-1R1 binding without signaling, and the decoy receptor IL-1R2, which traps cytokines without transmitting a signal [40]. The failure of these regulatory mechanisms leads to the pathological states targeted by modern pharmacotherapy.

### 2.3 Clinical relevance of IL-1 signaling in disease pathogenesis

Aberrant activation of the interleukin-1 (IL-1) signaling pathway serves as a central pathogenic mechanism across a broad spectrum of inflammatory disorders [41]. Sustained engagement of its downstream effectors—particularly the NF-κB and MAPK cascades—drives overproduction of key pro-inflammatory mediators, including IL-6, TNF-α, and COX-2, thereby contributing to progressive tissue injury and remodeling [42].

In rheumatoid arthritis (RA), IL-1 intensifies synovial inflammation and accelerates both cartilage matrix degradation and erosive bone loss [43]. These catabolic effects are mediated principally through induction of matrix metalloproteinases (MMPs) and stimulation of osteoclast differentiation and activity [44]. During acute gout flares, monosodium urate crystals act as danger-associated molecular patterns (DAMPs) that activate the NLRP3 inflammasome, leading to caspase-1-dependent maturation of pro-IL-1β and the subsequent amplification of acute neutrophilic inflammation [45]**.** Cryopyrin-associated periodic syndromes (CAPS), by contrast, arise from gain-of-function mutations in NLRP3 that result in constitutive inflammasome assembly, persistent IL-1β release, and recurrent systemic autoinflammatory episodes [46].

Chronically elevated IL-1β further contributes to the progression of several major non-communicable diseases [47]**.** In atherosclerosis, IL-1β compromises endothelial barrier integrity, promotes plaque instability, and heightens thrombotic risk [48]**.** In type 2 diabetes mellitus, it exerts direct cytotoxic effects on pancreatic β-cells, thereby impairing insulin secretion and exacerbating peripheral insulin resistance [49]**.** Within the tumor microenvironment, IL-1α released from necrotic cells functions as an endogenous alarmin that facilitates angiogenesis, stromal remodeling, and distant metastasis in malignancies such as colorectal cancer and lung cancer [50].

The multifaceted pathogenic contributions of IL-1 across these conditions provide a strong biological rationale for targeted therapeutic blockade [51]; and competitive inhibition of IL-1 receptor type I (IL-1R1) with recombinant antagonists such as anakinra, which blocks signaling initiated by both IL-1α and IL-1β isoforms [52]. These agents have demonstrated clear benefit in controlling autoinflammatory manifestations in CAPS, attenuating joint destruction in RA and gout, and—particularly with canakinumab—reducing cardiovascular events in patients with residual inflammatory risk [53]**.**

## 3 Biophysical constraints and advantages of evolving antibody scaffolds

The evolution of IL-1 therapeutics is intrinsically linked to the structural evolution of antibodies themselves. As depicted in Fig. 3, distinct modalities—Polyclonal, Monoclonal, and Single-Domain—offer unique biophysical profiles that dictate their application.

### 3.1 Polyclonal avidity and the Paradox of the Carrier Effect

Polyclonal antibodies (Fig. 3, Middle) represent the earliest form of immunological tool. Generated by immunizing animals (typically rabbits, goats, or sheep) with purified antigen, pAbs are heterogeneous mixtures of immunoglobulins that recognize multiple distinct epitopes on the IL-1 protein [54].

#### 3.1.1 Mechanism and utility

Due to their multi-epitope recognition, pAbs possess high avidity. Even if the antigen is partially denatured or buried, a pAb mixture is likely to contain clones that can bind. This establishes them as a primary diagnostic tool for techniques like Western Blotting, ELISA, and Immunohistochemistry (IHC), where sensitivity is paramount. In the Foundational Era (Fig. 4), these tools were instrumental in quantifying IL-1 levels in synovial fluid and identifying the presence of cytokines in diverse biological samples.

#### 3.1.2 The "Carrier Effect"

However, their therapeutic utility is severely limited by a phenomenon known as the "Carrier Effect." Research by Gunther Spohn [55] and colleagues demonstrated that certain anti-IL-1β pAbs can bind the cytokine in circulation without neutralizing it. Instead of clearing the cytokine, the antibody protects it from proteolytic degradation and renal filtration. The antibody effectively acts as a carrier protein, extending the serum half-life of IL-1β from minutes to days. This immune complex formation can paradoxically enhance bioactivity, acting as a slow-release reservoir of inflammation. In animal models, this led to exacerbated disease phenotypes, such as worsened arthritis or colitis. Furthermore, the batch-to-batch variability and high immunogenicity of animal-derived proteins preclude their use in chronic human therapy.

### 3.2 Pharmacokinetic profiles of the monoclonal IgG standard

The advent of hybridoma technology and subsequent humanization techniques enabled the broad clinical translation of monoclonal antibodies (Fig. 3, Left). These are bivalent IgG molecules (∼150 kDa) derived from a single B-cell clone, providing high target specificity for a single epitope [56].

#### 3.2.1 Structure and pharmacokinetics

The defining feature of mAbs is their Fc region [57,58]. This domain binds to the neonatal Fc receptor (FcRn) on endothelial cells, protecting the antibody from lysosomal degradation and recycling it back into circulation [59,60]. This mechanism grants mAbs a serum half-life of 2-3 weeks, enabling convenient dosing schedules (e.g., monthly subcutaneous injections) essential for chronic disease management.

#### 3.2.2 Mechanism of action

Clinically approved mAbs like canakinumab function via steric blockade. By binding to the receptor-interaction site on IL-1β with picomolar affinity, they physically occlude the interface required for IL-1R1 engagement. This high-affinity neutralization is the gold standard for systemic therapy. However, the large hydrodynamic radius of IgGs limits their passive diffusion [61]. In dense, fibrotic tissues—such as the capsular synovium or the desmoplastic stroma of a tumor—the local concentration of mAb may be insufficient to neutralize the high local cytokine load. This "tissue barrier" effect is a primary driver for the investigation of smaller antibody formats [62].

### 3.3 Superior tissue penetration of camelid and shark single-domain architectures

A significant structural advancement is the development of single-domain antibodies, or Nanobodies (Fig. 3, Right). Derived from the heavy-chain-only antibodies (HCAbs) found in Camelids (camels, llamas, alpacas) and cartilaginous fish, these molecules consist solely of the variable heavy domain (VHH) [63,64].

#### 3.3.1 Biophysical advantages

At approximately 15 kDa, nanobodies are one-tenth the size of a conventional IgG [[65], [66], [67]]. This size reduction confers several critical advantages.

• **Tissue Penetration:** Nanobodies exhibit rapid extravasation and superior diffusion coefficients, allowing them to penetrate dense tissues, necrotic cores, and even cross the blood-brain barrier more effectively than IgGs [68,69].
• **Cryptic Epitopes:** The antigen-binding loop (CDR3) of a VHH domain is often longer and more convex than that of a human VH-VL pair. This allows nanobodies to extend into clefts and active sites on the IL-1 molecule that are sterically inaccessible to the flat paratope of a standard antibody [70].
• **Stability:** VHH domains are highly soluble and thermally stable, resisting aggregation even under harsh conditions [71]. This robustness makes them ideal candidates for complex bioengineering applications, such as conjugation to hydrogels or nanoparticles, where standard proteins might denature [72].

#### 3.3.2 The rise of shark VNARs

While camelid VHHs are well-established, the Variable New Antigen Receptor (VNAR) domains from sharks—such as nurse sharks (*Ginglymostoma cirratum*) and whitespotted bamboo sharks (*Chiloscyllium plagiosum*)—are emerging as powerful alternatives. Kim et al. [73] demonstrated that sharks mount robust humoral responses, generating high-affinity libraries against targets like the SARS-CoV-2 RBD. Comparative studies by Barelle's group indicate that VNARs can exhibit superior affinity and stability profiles relative to equivalent VHH domains [74].

The potential of VNARs extends to oncology and diagnostics [75]. Lyu's group has successfully screened high-affinity VNARs targeting human VEGFR2 to induce anti-angiogenic effects [76], and IL-13Rα2 to inhibit glioma migration [77]. Additionally, anti-CD20 VNARs have shown effective cytotoxicity against lymphoma cells [78]. These findings highlight the dual-edged potential of VNARs: they can starve vascularized tumors via VEGFR2 blockade or exploit blood-brain barrier permeability to target gliomas. Thus, shark-derived scaffolds are emerging as a highly competitive alternative to camelids as a premier model organism for single-domain antibody discovery.

As summarized in Table 1, the unique structural features of shark-derived VNARs—including their exceptionally long CDR3 (up to 40 amino acids) and non-canonical disulfide bond networks (Types I–IV)—make them particularly well-suited for targeting complex inflammatory microenvironments (such as necrotic zones), enabling superior affinity and penetration toward cryptic epitopes.

**Table 1:** Structural comparison of conventional VH/VL (scFv/Fab), camelid VHH, and shark VNAR, and their advantages for IL-1 targeting.

Feature	Conventional VH/VL (scFv/Fab)	Camelid VHH	Shark VNAR	Advantage for IL-1 Targeting
Molecular weight	∼25–30 kDa	12–15 kDa	12–15 kDa	Superior tissue penetration
CDR number	6 (3 VH + 3 VL)	3	2 (CDR1 + CDR3)	Simplified paratope
CDR3 length range	9–15 aa	12–18 aa	10–40 aa (avg. 15–30+)	Better access to cryptic/hidden epitopes
Non-canonical disulfides	Rare	Common (CDR1-CDR3)	Highly variable (Types I–IV)	Enhanced thermal/structural stability
β-strands in framework	9–10	9	8	More compact scaffold
Epitope preference	Flat surfaces	Concave/flat	Cryptic/concave (deep pockets)	Ideal for necrotic/fibrotic IL-1 sites

## 4 Therapeutic validation across oncology, cardiovascular, and systemic rheumatologic pathologies

The clinical application of anti-IL-1 therapies has validated the distinct roles of IL-1α and IL-1β in human disease. As outlined in the **Clinical Translation Era** of Fig. 4, the development of specific inhibitors has transformed the management of several refractory conditions.

### 4.1 Modulation of the tumor microenvironment via the IL-1α alarmin axis

While IL-1β has historically garnered more attention, the targeting of IL-1α has revealed critical insights into sterile inflammation and paraneoplastic syndromes. The lead agent in this class is MABp1 (Bermekimab), a cloning-derived human monoclonal antibody [79,80].

#### 4.1.1 Metastatic colorectal cancer (mCRC)

In advanced cancer, patients often suffer from cachexia, fatigue, and anorexia—symptoms driven by a systemic inflammatory state [81,82]. IL-1α, released from the necrotic tumor core, acts on the central nervous system to alter metabolism and appetite. A pivotal Phase III randomized clinical trial demonstrated the efficacy of MABp1 in this context. In patients with metastatic colorectal cancer refractory to standard chemotherapy, MABp1 treatment resulted in a significant improvement in a composite endpoint of lean body mass and symptom control. Specifically, 33% of patients in the treatment arm achieved symptom relief (reduction in pain, fatigue, and anorexia) compared to only 19% in the placebo group [83]. This study was a landmark proof-of-concept that targeting the "alarmin" function of IL-1α could modify the host response to malignancy, improving quality of life even in late-stage disease.

#### 4.1.2 Hidradenitis suppurativa (HS)

HS is a debilitating dermatological condition characterized by painful, draining lesions in apocrine gland-bearing skin [[84], [85], [86]]. The pathology is driven by a feedback loop where IL-1α stimulates the overproduction of human beta-defensin 2 (hBD-2) [87,88] in skin lesions. Clinical investigations have shown that MABp1 can disrupt this axis. By neutralizing IL-1α, the antibody reduces the inflammatory burden and promotes the healing of deep, tunneling wounds, offering an alternative for patients who do not respond to TNF-α inhibitors like adalimumab.

### 4.2 Systemic neutralization of IL-1β in autoinflammatory and ischemic pathologies

Canakinumab (ACZ885), a high-affinity anti-IL-1β mAb [89], serves as a primary hallmark of the "monoclonal era." Its clinical development has systematically validated the role of IL-1β across a spectrum of diseases [47].

#### 4.2.1 Autoinflammatory syndromes (CAPS)

The most direct evidence for IL-1β pathogenicity comes from Cryopyrin-Associated Periodic Syndromes (CAPS) [90]. These rare genetic disorders—Familial Cold Autoinflammatory Syndrome (FCAS), Muckle-Wells Syndrome (MWS), and Neonatal-Onset Multisystem Inflammatory Disease (NOMID/CINCA) [91,92]—are caused by gain-of-function mutations in the NLRP3 gene, leading to constitutive inflammasome activation [93]. Patients suffer from recurrent fevers, urticaria-like rashes, and severe arthropathy. Canakinumab serves as a "replacement" for the body's failed regulatory mechanisms, inducing rapid and sustained remission of fever and rash within hours of administration. It is currently the standard of care, highlighting the power of precision medicine in monogenic diseases.

#### 4.2.2 Cardiovascular inflammation (The CANTOS trial)

Atherosclerosis is fundamentally an inflammatory process involving cholesterol crystals, which—like uric acid crystals in gout—activate the NLRP3 inflammasome in plaque macrophages [94,95]. The CANTOS trial (Canakinumab Anti-inflammatory Thrombosis Outcome Study) provided pivotal clinical evidence in cardiovascular medicine [96,97]. It enrolled over 10,000 post-myocardial infarction patients with persistently elevated high-sensitivity C-reactive protein (hs-CRP) [98]. Treatment with canakinumab significantly reduced the rate of recurrent cardiovascular events (MI, stroke, cardiovascular death) independent of lipid lowering [99,100]. This demonstrated, for the first time in a large-scale trial, that targeting inflammation per se (specifically the IL-1β/IL-6 axis) confers cardiovascular protection [101]. However, the trial also noted a dose-dependent increase in fatal infections, underscoring the risks of systemic innate immune suppression.

#### 4.2.3 Gouty arthritis

In gout, monosodium urate crystals act as the "Signal 2″ for IL-1β release [102,103]. Canakinumab has shown remarkable efficacy in terminating acute flares and preventing recurrences in patients contraindicated for NSAIDs or colchicine [104,105]. Its long half-life provides sustained protection, although cost and infection risks limit its use to refractory cases.

#### 4.2.4 Systemic juvenile idiopathic arthritis (sJIA) and adult-onset Still's disease (AOSD)

Beyond monogenic autoinflammatory syndromes and metabolic inflammation, the clinical application spectrum of IL-1 neutralization is perhaps most transformative in complex, polygenic rheumatologic diseases, notably systemic juvenile idiopathic arthritis (sJIA) and its adult continuum, adult-onset Still's disease (AOSD). These severe conditions are characterized by spiking fevers, evanescent rashes, and destructive arthritis, driven by a profound dysregulation of the innate immune system where IL-1β functions as the central pathological node [106,107]. Prior to the advent of IL-1 blockade, sJIA and AOSD were associated with significant morbidity, growth retardation in children, and a heavy reliance on high-dose glucocorticoids [108]**.**

The introduction of IL-1 inhibitors represents a paradigm shift in managing the Still's disease continuum [109]**.** Extensive evidence from both clinical trials and real-world registries confirms that early targeted intervention with canakinumab or anakinra rapidly resolves systemic inflammation; crucially, it alters the disease course by mitigating debilitating joint destruction and reducing the incidence of life-threatening macrophage activation syndrome (MAS) [110,111]**.**Importantly, early introduction of IL-1 blockade in sJIA has been shown to induce a "window of opportunity," potentially altering the disease course and allowing for drug-free remission [112]. This significant efficacy underscores IL-1β as a central mediator in Still's disease, explicitly expanding the therapeutic horizon of IL-1 targeted therapies well beyond cardiovascular and oncological boundaries and validating its role in broadly suppressing pathological innate immune hyperactivation.

### 4.3 Dual blockade with bispecific antibodies targeting IL-1α and IL-1β

Although mono-specific antagonists have demonstrated definite clinical efficacy, dual neutralization of IL-1α and IL-1β is emerging as a crucial strategy to overcome limitations arising from their potential compensatory mechanisms. A representative agent in this domain, lutikizumab (ABT-981), utilizes a dual-variable domain immunoglobulin (DVD-Ig) format capable of simultaneously binding and neutralizing both IL-1α and IL-1β with high affinity [113]. Preclinical data indicate that in osteoarthritis (OA) models, ABT-981 exhibits significantly superior efficacy compared to mono-specific inhibitors in attenuating cartilage matrix degradation and mitigating synovial inflammation. Furthermore, alternative dual-target interventions, such as single-chain bispecific antibodies directed against IL-1β and IL-17A, have demonstrated synergistic effects in rheumatoid arthritis (RA) models, effectively interrupting the inflammatory cascade [114]. These advancements suggest that for pathological processes co-mediated by IL-1α and IL-1β, dual-blockade strategies hold the potential to deliver superior disease-modifying effects, offering a critical framework for the development of next-generation therapeutics targeting the IL-1 pathway.

### 4.4 Synergistic approaches: Combinatorial strategies with IL-1 blockade

While monotherapy has provided critical insights into IL-1 biology, the clinical reality of complex oncological and hematological conditions increasingly necessitates combinatorial strategies. In the tumor microenvironment (TME), IL-1β plays a pivotal role in maintaining immunosuppression by recruiting myeloid-derived suppressor cells (MDSCs) and promoting angiogenesis [115]. Consequently, combining IL-1 blockade with immune checkpoint inhibitors (ICIs), such as anti-PD-1/PD-L1 antibodies, has emerged as a rational strategy to overcome immunotherapy resistance [116]**.** Although the phase III CANOPY-1 trial—which evaluated canakinumab in combination with pembrolizumab and platinum-based chemotherapy in advanced non-small cell lung cancer (NSCLC)—did not meet its primary overall survival endpoints, it underscored the complexity of systemic inflammation and suggested that specific patient subgroups with elevated baseline inflammatory biomarkers may still derive clinical benefit from this dual blockade [117]**.**

Beyond enhancing solid tumor efficacy, IL-1 targeted therapies have become an indispensable combinatorial adjunct in cellular immunotherapy. Chimeric antigen receptor (CAR) T-cell therapy is frequently complicated by severe cytokine release syndrome (CRS) and immune effector cell-associated neurotoxicity syndrome (ICANS) [118]. Recent clinical evidence demonstrates that the prophylactic or early therapeutic administration of anakinra (IL-1Ra) can effectively mitigate severe ICANS and tocilizumab-refractory CRS without compromising the cytotoxic anti-tumor efficacy of the CAR-T cells [119]**.** This dual utility—potentiating the efficacy of ICIs while dampening the life-threatening toxicities of engineered T-cells—highlights a critical translational paradigm: treating IL-1 not merely as a primary target, but as a core immunomodulatory node to optimize broader immunotherapeutic regimens.

## 5 Advanced biomaterials for tissue-specific delivery, tracking, and immunomodulation

As outlined in Fig. 4, the field is advancing toward the "Bioengineering Era" to overcome the biophysical constraints of conventional monoclonal antibodies. While mAbs serve as the clinical standard, their therapeutic utility is increasingly circumscribed by two critical limitations: a large hydrodynamic radius that hinders effective diffusion into necrotic tissue cores, and a systemic mode of action that carries the liability of broad immunosuppression. To overcome these biophysical limitations, the focus has turned to the convergence of structural biology and materials engineering, leveraging the synergy between nanobody technology and advanced biomaterials to create modular, tissue-specific solutions.

### 5.1 Overcoming ischemic barriers with nanobodies

Myocardial Infarction (MI) presents a unique pharmacological challenge [120]. The onset of ischemia causes massive cardiomyocyte necrosis [121,122], releasing IL-1α and subsequently triggering an intense IL-1β-mediated inflammatory response. This hyperinflammatory cytokine cascade clears debris but, if unchecked, leads to fibrosis, adverse ventricular remodeling, and heart failure.

Standard mAbs are too large to diffuse efficiently into the dense, poorly perfused infarct zone. In contrast, Nanobodies (VHHs), with their small hydrodynamic radius (∼2.5 nm vs ∼5 nm for IgG), exhibit superior diffusion kinetics [123,124]. They can permeate the extracellular matrix of the injured myocardium to reach high local concentrations. Furthermore, their modular nature allows for the construction of multivalent formats that can bind multiple cytokines simultaneously or engage specific tissue markers for retention.

### 5.2 Intelligent ROS-responsive hydrogels for localized therapeutic rescue

To further refine this approach, researchers are engineering stimuli-responsive nanocarriers capable of sensing and responding to pathological triggers. A salient example from recent literature is the development of Reactive Oxygen Species (ROS)-responsive hydrogels [125]. The microenvironment of an acute Myocardial Infarction (MI) is defined by severe oxidative stress and a massive elevation in ROS [126]. Bioengineers have exploited this pathological hallmark by synthesizing hydrogels cross-linked with ROS-cleavable chemistries, such as thioketal or boronic ester bonds [127].

While the small hydrodynamic radius (∼15 kDa) of nanobodies facilitates exceptional tissue penetration, it paradoxically renders them susceptible to rapid renal clearance—falling well below the ∼60 kDa glomerular filtration threshold—and proteolytic degradation in vivo [128]. Consequently, unformulated nanobodies typically exhibit a systemic half-life restricted to mere minutes or hours, severely limiting their sustained therapeutic efficacy [129]. The integration of these single-domain antibodies into a stimuli-responsive hydrogel matrix fundamentally resolves these pharmacokinetic liabilities.

Mechanistically, the three-dimensional polymeric network acts as a highly localized therapeutic depot [130]. Its dense cross-linked architecture exerts strong steric hindrance that physically restricts the diffusion of host proteolytic enzymes into the matrix, thereby shielding the encapsulated nanobodies from premature enzymatic degradation [131]. Furthermore, the tunable mesh size of the hydrogel effectively restricts the passive diffusion of the nanobodies, preventing their premature systemic dissemination [132]. In the proposed system, anti-IL-1β nanobodies are sequestered within the hydrogel matrix. Upon injection into the pericardial space or myocardium, the intact scaffold anchors the nanobodies within the healthy tissue margin [133]. However, direct contact with the hyper-oxidative infarct zone triggers the targeted degradation of the ROS-cleavable bonds [134]. This environment-responsive transition shifts the delivery kinetics from diffusion-controlled to degradation-controlled, achieving a synchronized, "on-demand" release of the nanobody directly at the nidus of inflammation, perfectly matching the spatiotemporal profile of the disease [135]. This bioengineered precision resolves the central dilemma of previous systemic therapies. By maintaining a high therapeutic index locally, the system effectively neutralizes the cytokine surge responsible for adverse remodeling. Simultaneously, it ensures systemic safety: because the nanobodies are released locally and possess a short systemic half-life due to rapid renal clearance, circulating drug levels remain negligible. This design significantly mitigates the risks of systemic immunosuppression and opportunistic infection that confounded earlier trials like CANTOS. Validated by preliminary animal studies demonstrating preserved ventricular function and reduced scar burden, this "immuno-regenerative" strategy epitomizes the cutting-edge innovations of the "Bioengineering Era" illustrated in Fig. 4.

### 5.3 Expanding the biomaterial repertoire: Nanocarriers and theranostic platforms

While macroscopic hydrogels excel at establishing localized physical depots, the broader field of biomaterials offers complementary strategies for systemic administration with cellular-level precision. Beyond stimulus-responsive cross-linking, advanced particulate systems—such as poly(lactic-co-glycolic acid) (PLGA) nanoparticles and lipid-based vesicles—are being extensively engineered for the precise delivery and sustained release of IL-1 targeted biologics [136]. These nanocarriers effectively encapsulate fragile protein antagonists or nanobodies, shielding them from premature enzymatic degradation and rapid renal clearance [137]. By tuning the polymer molecular weight and lactide-to-glycolide ratio, researchers can engineer these platforms to exhibit predictable, sustained release kinetics, transitioning therapy from frequent burst-dosing to steady-state immunomodulation [138].

Furthermore, these particulate biomaterials are critical for therapeutic potentiation through active targeting. By functionalizing the surface of nanocarriers with specific ligands—such as hyaluronic acid to engage CD44 receptors overexpressed on activated macrophages—biomaterials can facilitate receptor-mediated endocytosis [139]. This targeted endosomal delivery directs the therapeutic payload precisely to the cellular source of the inflammasome cascade, maximizing local efficacy while minimizing off-target receptor blockade in healthy tissues [140].

Simultaneously, the integration of imaging modalities with these delivery vehicles has driven the emergence of "theranostics" (therapy + diagnostics). A major clinical challenge in treating sterile inflammation is the inability to non-invasively monitor disease activity and drug biodistribution. Biomaterials bridge this gap. By conjugating anti-IL-1 nanobodies to superparamagnetic iron oxide nanoparticles (SPIONs) or radiolabeling polymeric carriers, researchers have created theranostic agents detectable via MRI or Positron Emission Tomography (PET) [141]. This dual-functionality allows clinicians to visually track the accumulation of the drug at localized inflammatory foci (e.g., vulnerable atherosclerotic plaques or occult arthritic joints) in real-time. Ultimately, the integration of imaging and tracking capabilities ensures that potent immunomodulators are deployed strictly when and where the target cytokine is actively expressed, establishing a feedback loop that defines the future of precision immunology [142].

## 6 Conclusion and future perspectives

The trajectory of IL-1 research—from the initial cloning of a fever-inducing factor to the sophisticated engineering of stimuli-responsive hydrogels—mirrors the maturation of modern immunology. The field has advanced from early polyclonal detection methods of the "Foundational Era" to precision therapeutic tools capable of targeting complex inflammatory microenvironments. This evolution has been guided by a deepening understanding of the functional dichotomy between IL-1α, the locally acting alarmin found in necrotic microenvironments, and IL-1β, the systemic pyrogen driven by the inflammasome.

While monoclonal antibodies like canakinumab defined "Generation 2.0″ therapeutics—successfully managing systemic pathologies such as CAPS, atherosclerosis, and gout—they have likely reached their maximum therapeutic potential. The large hydrodynamic size of IgGs restricts their penetration into dense, fibrotic tissues, and their systemic mode of action carries inherent risks of immunosuppression. Consequently, the field is pivoting toward a "Generation 3.0″ paradigm: the Bioengineering Era, characterized by modular, tissue-specific, and environment-responsive interventions.

Central to this shift is the integration of single-domain antibodies with smart biomaterials. By encapsulating nanobodies within ROS-responsive hydrogels, we can now achieve "on-demand" release, concentrating therapeutic power specifically at ischemic sites like the infarcted heart while sparing the systemic immune system. However, the clinical translation of these smart platforms necessitates surmounting specific biophysical barriers. In dense, inflamed, or fibrotic microenvironments—such as the post-myocardial infarction scar—passive diffusion is profoundly restricted by excessive extracellular matrix (ECM) deposition [143].Consequently, the physical dimensions and degradation kinetics of hydrogel formulations are paramount to their efficacy. Particulate carriers or degraded hydrogel fragments exceeding 200 nm are typically sequestered in the perivascular space or rapidly cleared by the reticuloendothelial system. In contrast, sub-100 nm geometries exhibit optimal permeation and retention within injured myocardial tissue [144]. Parallel to overall formulation size, the internal mesh size of the hydrogel must be precisely calibrated against its therapeutic payload. A structural mismatch between the polymer pore network and the hydrodynamic radius of the nanobody (approx. 2.5 nm) can precipitate either premature burst release into the systemic circulation or incomplete localized delivery [145]. Tuning these geometric and kinetic parameters to balance matrix stability with efficient payload diffusion remains a critical imperative for advancing these biomaterial therapies [146]. Furthermore, the source of these biological tools is expanding. While camelids have long been the standard for single-domain antibody discovery, the whitespotted bamboo shark is emerging as a superior model organism. As a key species in immune evolution, these cartilaginous fish possess a unique adaptive immune system that yields VNARs with exceptional stability and affinity.

Recent breakthroughs have validated the versatility of these shark-derived binders: they have been engineered to starve tumor angiogenesis via VEGFR2 blockade, inhibit glioma invasion by targeting IL-13Rα2, and deliver cytotoxic payloads to CD20-positive lymphomas. This evolutionary edge suggests that *Chiloscyllium plagiosum* VNARs may soon supplant camelid VHHs as the preferred scaffold for unraveling complex disease mechanisms.

Looking forward, the rational engineering of next-generation IL-1 therapeutics is poised to overcome the structural limitations of current monoclonal platforms. Rather than relying solely on monovalent neutralization, future designs will increasingly leverage biparatopic and multispecific architectures. For example, linking two distinct VHH domains to create biparatopic nanobodies can synergistically increase binding avidity and neutralize cryptic epitopes more potently than conventional bivalent formats [147,148]. Furthermore, managing complex autoinflammatory conditions requires dismantling redundant cytokine networks. The development of bispecific antibodies—such as MAS825, which co-targets IL-1β and IL-18 (both inflammasome-derived pyrogens)—offers a robust strategy to prevent the compensatory immune escape mechanisms that often circumvent monotherapy [149].

Crucially, the clinical translation of these engineered biologics depends heavily upon their integration with advanced biomaterials to resolve the persistent dilemma of systemic toxicity. Conventional systemic administration of cytokine inhibitors often results in a narrow therapeutic window, compromised by severe off-target immunosuppression (as highlighted by the fatal infection risks in the CANTOS trial) [150]. By conjugating these biologics to stimuli-responsive hydrogels or polymeric nanocarriers, therapeutics are shielded from premature enzymatic degradation and deployed specifically within the inflammatory nidus [151]. This localized, sustained-release mechanism broadens the therapeutic index significantly: it maintains maximal receptor-occupancy within ischemic or fibrotic tissues while keeping systemic drug concentrations negligible, thereby maximizing therapeutic efficacy and drastically mitigating the risk of opportunistic infections [152]**.**

To fully realize this paradigm of precision immunomodulation, the field must advance along three concrete directions.

1. **AI-Driven Structural Engineering:** Integrating artificial intelligence (AI) and machine learning algorithms with structural biology to accelerate the *de novo* design and affinity maturation of multispecific nanobodies with optimized thermodynamic stability [153].
2. **Theranostic Nanosystems:** Developing dual-function theranostic platforms that pair real-time molecular imaging (e.g., immuno-PET) of IL-1 inflammatory hotspots with the precise, environment-triggered release of therapeutic payloads [154].
3. **Clinical Translation for Chronic Niches:** Expanding application from acute models to chronic localized conditions, such as employing long-acting, intra-articular nanobody-hydrogel matrices for severe osteoarthritis, thereby redefining targeted joint preservation.

Ultimately, by treating inflammation as a dynamic, spatiotemporal event rather than a static systemic condition, the convergence of structural immunology and materials science will yield therapeutic interventions as precise as the immune response itself.

## CRediT authorship contribution statement

**Lan Yang:** Writing – original draft, Writing – review & editing. **Guodong Chen:** Formal analysis, Funding acquisition, Resources, Writing – review & editing. **Zhengbing Lyu:** Data curation, Funding acquisition, Project administration.

## Declaration of competing interest

The authors declare that they have no known competing financial interests or personal relationships that could have appeared to influence the work reported in this paper.

## Data Availability

Data will be made available on request.
